# Using machine learning involving diagnoses and medications as a risk prediction tool for post-acute sequelae of COVID-19 (PASC) in primary care

**DOI:** 10.1186/s12916-025-04050-w

**Published:** 2025-04-30

**Authors:** Seika Lee, Marta A. Kisiel, Pia Lindberg, Åsa M. Wheelock, Anna Olofsson, Julia Eriksson, Judith Bruchfeld, Michael Runold, Lars Wahlström, Andrei Malinovschi, Christer Janson, Caroline Wachtler, Axel C. Carlsson

**Affiliations:** 1 Occupational and Environmental Medicine, Department of Medical Sciences, Uppsala University, Uppsala, Sweden; 2 Division of Immunology and Respiratory Medicine, Department of Medicine Solna, Karolinska Institutet, Stockholm, Sweden; 3 Department of Respiratory Medicine and Allergy, and Center for Molecular Medicine, Karolinska University Hospital Solna, Solna, Sweden; 4 Division of Biostatistics, Institute of Environmental Medicine, Karolinska Institutet, Stockholm, Sweden; 5 Division of Infectious Diseases, Department of Medicine Solna, Karolinska Institutet, Stockholm, Sweden; 6 Department of Infectious Diseases, Karolinska University Hospital, Stockholm, Sweden; 7 Centre for Psychiatry Research, Karolinska Institutet, Stockholm, Sweden; 8 Clinical Physiology, Department of Medical Sciences, Uppsala University, Uppsala, Sweden; 9 Respiratory Medicine, Department of Medical Sciences, Uppsala University, Uppsala, Sweden; 10 Department of Neurobiology, Care Sciences and Society, Division of Family Medicine and Primary Care, Karolinska Institutet, Huddinge, Sweden; 11 Academic Primary Health Care Centre, Region Stockholm, Stockholm, Sweden

**Keywords:** Post-acute sequelae of COVID- 19 (PASC), COVID- 19, Primary care, Machine learning, Stochastic gradient boosting

## Abstract

**Background:**

The aim of our study was to determine whether the application of machine learning could predict PASC by using diagnoses from primary care and prescribed medication 1 year prior to PASC diagnosis.

**Methods:**

This population-based case–control study included subjects aged 18–65 years from Sweden. Stochastic gradient boosting was used to develop a predictive model using diagnoses received in primary care, hospitalization due to acute COVID- 19, and prescribed medication. The variables with normalized relative influence (NRI) ≥ 1% showed were considered predictive. Odds ratios of marginal effects (OR_ME_) were calculated.

**Results:**

The study included 47,568 PASC cases and controls. More females (*n* = 5113) than males (*n* = 2815) were diagnosed with PASC. Key predictive factors identified in both sexes included prior hospitalization due to acute COVID- 19 (NRI 16.1%, OR_ME_ 18.8 for females; NRI 41.7%, OR_ME_ 31.6 for males), malaise and fatigue (NRI 14.5%, OR_ME_ 4.6 for females; NRI 11.5%, OR_ME_ 7.9 for males), and post-viral and related fatigue syndromes (NRI 10.1%, OR_ME_ 21.1 for females; NRI 6.4%, OR_ME_ 28.4 for males).

**Conclusions:**

Machine learning can predict PASC based on previous diagnoses and medications. Use of this AI method could support diagnostics of PASC in primary care and provide insight into PASC etiology.

**Supplementary Information:**

The online version contains supplementary material available at 10.1186/s12916-025-04050-w.

## Background

Coronavirus disease 2019 (COVID- 19), caused by the severe acute respiratory syndrome coronavirus 2 (SARS-CoV- 2), caused a global emergency from 2020 to 2022. Although the widespread outbreak subsided in 2022, infections continue to occur [1]. The majority of those infected recover, but some individuals experience persistent symptoms known as post-acute sequelae of COVID- 19 (PASC). The World Health Organization defines PASC as illness that occurs within 3 months of COVID- 19 infection and cannot be explained by alternative diagnoses [2]. PASC is characterized by a wide range of symptoms. It can significantly impair daily functioning and is currently a common cause of sick leave [3–6]. At the time being, there are no objective diagnostic tests or accessible biomarkers for the condition, and the diagnosis is based on organ dysfunction, various symptoms and their duration. Nor has the pathophysiology of PASC been clearly elucidated [7, 8]. Finding diagnostic tools and understanding the etiology of the condition is a challenge; such developments might potentially improve management and outcomes for those affected.

There is growing evidence indicating that females have a higher risk of developing PASC compared to males [9, 10]. Other identified risk factors for PASC include belonging to certain demographic groups (e.g., females aged 35–50 years and socioeconomically deprived individuals), having pre-existing health conditions, such as obesity and cardiovascular disease, experience of more severe acute illness, and being unvaccinated [9, 10]. These risk factors have mainly been based on follow-up studies of hospitalized patients [11, 12] and questionnaire data [13–16]. Epidemiological studies on PASC are difficult to interpret and combine, because inclusion criteria, diagnostic criteria, and methodologies vary [10]. A Swedish study based on register data from secondary care found that symptoms of dyspnea and fatigue, and abnormal pulmonary imaging or findings, were associated with a PASC diagnosis [17]. Research to identify subtypes of PASC has yielded variable results thus far [4, 18]. Little is known about individuals who have developed PASC and were not hospitalized, were only in contact with primary care, or were not even in contact with healthcare in regard to COVID- 19.

Recently, machine learning methods have been applied to structure large amounts of patient data from multiple sources, improving the identification of chronic diseases, including new-onset diabetes [19], cardiovascular disease [20–22] and cancer [23, 24]. In the COVID- 19 context, machine learning models have been tested to describe the nature of PASC in terms of demographic features, symptom severity, and duration [25, 26]. A few previous studies have used machine learning to predict risk factors associated with PASC [27, 28]. For example, two studies demonstrated that the majority of individuals with PASC were female, with severe acute COVID- 19 and comorbidities including depression, type 2 diabetes, chronic kidney disease, and chronic pulmonary disease [27, 28]. In the context of PASC, machine learning outperforms traditional statistical models when predictive accuracy is the main goal because it can capture non-linear relationships and complex interactions between variables. Machine learning uses adaptive complex relationship through algorithms in our case thousands of decision trees, that perform better than variables in regression models that often have problems with collinearity [25].

Early studies during the pandemic predominantly used traditional statistics, such as logistic regression to identify predictors and risk factors of PASC [29]. Although, logistic regression is better for interpreting and understanding a hypothetical relationship that may be causal, introduces potential biases due to its simplicity, limited ability to capture complex interactions when the number of variables increase, and challenges in handling missing data [25, 30].These shortcomings are particularly significant for PASC, a condition with heterogeneous and overlapping symptoms. In contrast, machine learning demonstrates robust performance by leveraging data-driven feature selection and capturing non-linear relationships, enabling robust modeling of complex interactions among predictors [25, 30] underscored the potential of machine learning to identify novel, unexpected predictors in multifaceted conditions like PASC, where traditional models may falter. However, despite these advantages, the clinical relevance of machine learning for PASC remains underexplored [26].

Our study builds on prior research by applying stochastic gradient boosting (SGB), a machine learning method well-suited for analyzing high-dimensional data and identifying significant predictors. While earlier studies have predominantly focused on hospitalized populations or secondary care data, our work incorporates primary care diagnoses and prescription medications, offering a broader perspective on PASC predictors [25]. This approach highlights the potential of machine learning in diverse healthcare settings and may provide insights into the complex nature and potential drivers of PASC.

One of the machine learning-methods that can be used to predict medical conditions is stochastic gradient boosting (SGB) [25]. This technique is well-suited for analyzing high-dimensional datasets, as it can incorporate numerous variables while capturing complex, non-linear interactions among predictors. Unlike traditional statistical models, such as logistic regression, which often rely on linear assumptions and struggle with multi-collinearity or missing data, SGB is robust against these limitations. It employs iterative learning to minimize errors and improve prediction accuracy by combining the strengths of multiple weak learners (decision trees).

Moreover, SGB offers the capability to rank variables by their predictive importance, enabling a nuanced understanding of which factors contribute most to the outcome. This attribute is especially critical in multifaceted conditions like PASC, where predictors may interact in unexpected ways. Previous applications of SGB in our research demonstrated its effectiveness in identifying risk factors for chronic diseases, including colorectal cancer [23, 31] and diabetes [19]. These studies underline the suitability of SGB for modeling complex relationships in healthcare data, making it an ideal choice for exploring PASC predictors using primary care data and prescription history.

Given this background and knowledge gaps, we sought to determine whether the application of a machine learning model, SGB, could predict risk factors of PASC diagnosis. The model included all diagnoses from primary healthcare (PHC) consultations and dispensed prescribed medication during the year before PASC diagnosis. Previous hospitalization due to acute COVID- 19 before PASC diagnosis was also used in the model. The VAL database from Region Stockholm, which encompasses register data from primary care settings, was thought to be suitable. We hypothesized that this machine learning tool could be used as diagnostic support in primary care settings and identify predictors that could potentially play a role in the etiology of PASC.

## Methods

### Study design

This population-based case–control study encompassed subjects 18–65 years old who were registered at PHC centers (PHCCs) in the Stockholm Region in Sweden. The Stockholm Region is the largest metropolitan area in Sweden and has a total population of 2.5 million residents [32]. Register data for this study were gathered from the VAL database, which includes all registered diagnoses based on the International Classification of Diseases, Tenth Revision (ICD- 10), and all dispensed prescription drugs based on their Anatomical Therapeutic Chemical (ATC) code [33].

### Study population

The study cases included all individuals who had received the diagnosis post-COVID condition, unspecified (PASC, ICD- 10: U09.9) in any healthcare setting between 2020 and 2022. Each case was matched by age and sex with up to five controls who had not been diagnosed with PASC during the study period. Data on all diagnoses from physician consultations at PHCCs in Region Stockholm and dispensed prescribed medications were collected. We used prescribed medication as a proxy for chronic conditions. Hospitalization due to COVID- 19 before PASC diagnosis was also included.

Subjects who did not seek healthcare during the study period were excluded because they lacked data on visits and diagnoses, which are essential variables for the analysis. Including such individuals would not allow for meaningful comparisons between those with and without COVID- 19, as their absence from the healthcare system renders them non-contributory to predictive modeling. While we recognize that this exclusion introduces selection bias and limits the generalizability of our findings to those who engage with healthcare services, it was a necessary step to ensure the study’s objectives could be met. Future studies incorporating community-level data or self-reported health metrics could address this limitation and provide a different view of the population.

It was assumed that most of the population in Region Stockholm and the individuals in this study had had a COVID- 19 infection and were vaccinated for COVID- 19. Therefore, the diagnoses including COVID- 19 (ICD- 10: U07, U08), immunization against COVID- 19 (ICD- 10: U11) and adverse effects of COVID- 19 vaccines (ICD- 10: U12) were not included as predictors.

### Variables

We collected age at PASC diagnosis, sex, diagnoses (ICD-10 codes) from PHCCs, and dispensed prescribed medications reported during the 12 months prior to the index date (PASC diagnosis date). ICD codes for chronic diseases and conditions representing similar clinical features were merged into common clinical groups in accordance with previous studies (for further information see Additional File: Table 1). All other ICD codes were used as three-character codes, except Postviral fatigue, ICD code G93.3, which was deemed to have particular clinical relevance. It was therefore used as a four-character code, see Additional File: Table 1. A similar approach was used for ATC codes. All ATC-codes were one letter and two digits, and to distinguish medications of particular interest, they were in higher resolution, see Additional File: Table 2.

**Table 1:** Demographic characteristics of PASC cases and controls

Characteristic	PASC (***n*** = 7928)	Controls (***n*** = 39,640)
Sex, ***n***		
Male	2815	14,075
Female	5113	25,565
Age, years, mean		
Male	48	49
Female	47	47

*PASC*, post-acute sequelae of COVID- 19

**Table 2:** Confusion matrix for predicting presence of PASC among females and males in the test dataset using the stochastic gradient boosting model created from the training dataset

	Observed		
Predicted	No PASC	PASC	Total
Females (*n* = 9203)			
No PASC	5863	347	6210
PASC	1806	1187	2993
Total	7669	1534	9203
Males (*n* = 5067)			
No PASC	3505	173	3678
PASC	711	678	1398
Total	4216	851	5067

In females, predictions were based on 15,377 trees, with sensitivity 0.774 and specificity 0.765. In males, predictions were based on 11,221 trees, with sensitivity 0.797 and specificity 0.831

*PASC*, post-acute sequelae of COVID- 19

### Statistical methods

This study used the SGB technique for data analysis, an effective form of AI formerly utilized in similar research [34]. It has previously been applied by our research group to analyze factors influencing lung and colorectal cancer risk [23], and more recently diabetes and hypertension in primary care [19, 35]. The SGB model employed in this study is inherently capable of handling missing data by incorporating them as a separate category in the model. This feature ensures that individuals with incomplete data are not excluded from the analysis. The models were developed for males and females separately. For each of the two sets of training data, diagnoses and medications with at least 50 occurrences were selected. The optimal number of trees to use for prediction was estimated using tenfold cross-validation to ensure model robustness and prevent overfitting. Other hyperparameters, such as learning rate, maximum depth, and subsampling rate, were chosen based on prior studies by our group [19, 31], and validated for this dataset to align with best practices. This approach ensures that the parameters were both evidence-based and suitable for the current study context.

In this study, the top 2000 most common diagnoses registered in primary care were used for all 47,568 individuals. All dispensed drugs prescribed in primary and secondary care were included in the model. The diagnostic codes issued, and medications prescribed during the year before index date were used as predictors. By the use of this model, this resulted in 78 diagnoses and 52 medications for males and 125 diagnoses and 69 medications for females with at least 50 occurrences.

Next, the dataset was divided by sex, resulting in a group of 30,678 females and a group of 16,890 males. Applying a training-test approach for each group, we created a randomly 48 selected training set for each sub-dataset. Thus, 70% of the cases (*n* = 3579 females and *n* = 1964 males) with their matched controls (*n* = 21,475 females and *n* = 11,823 males) were used for training the SGB model. The remaining 30% of the cases (*n* = 1534 females and *n *= 851 males) with their controls (*n* = 9203 females and *n *=5067 males) were used for evaluating the model’s performance. The proportions of individuals with PASC were equal in the training and test datasets.

The performances of the final models were evaluated using area under the receiver operator characteristics (ROC) curve, sensitivity, and specificity. The SGB model was then applied to each test dataset to obtain patient-specific probabilities of being diagnosed with PASC. The probability that maximized the sum of sensitivity and specificity was used as a cut-off value such that patients with a probability higher than this cut-off were classified as being diagnosed with PASC. The results are presented in a confusion matrix, with the performance of the prediction given by sensitivity and specificity. The ROC curve shows the trade-off between true positive rate (sensitivity) and false positive rate (1—specificity) at various thresholds. The area under the curve (AUC, ranging from 0 to 1) summarizes the overall accuracy of the model. An AUC of 1 indicates perfect prediction and 0 no better than random prediction. These metrics provide valuable insights into the model's ability to distinguish between positive and negative instances.

From the SGB model, we obtained a ranking of the diagnoses most often related to the PASC diagnosis, presented as the normalized relative influence (NRI) with a corresponding odds ratio of marginal effects (OR_ME_) for being diagnosed with PASC. Based on our previous studies with the machine learning SGB [19, 35], we assumed 1% NRI as our cut-off threshold for clinically relevant diagnoses and prescribed medications. For each diagnosis, the odds ratio was calculated using the probability of being diagnosed with PASC, obtained by integrating out all other variables from the model using the weighted tree traversal method [34].

The analyses were performed using R version 4.2.1 [36].

## Results

### Study population

In total, there were 47,568 study subjects and controls, of whom 39,640 were controls matched by age and sex with the post-acute sequelae of COVID- 19 (PASC) cases. There were more females (*n* = 5113) than males (*n* = 2815) diagnosed with PASC between 2020 and 2022 (Table 1). The data for PASC cases and controls were divided into training and test datasets. The training dataset for females encompassed 21,475 subjects, whereas the test dataset for females encompassed 9203 subjects. The training and test datasets for males included 11,823 and 5067 subjects, respectively.

### Predictive ability of the SGB model

**Fig. 1:**
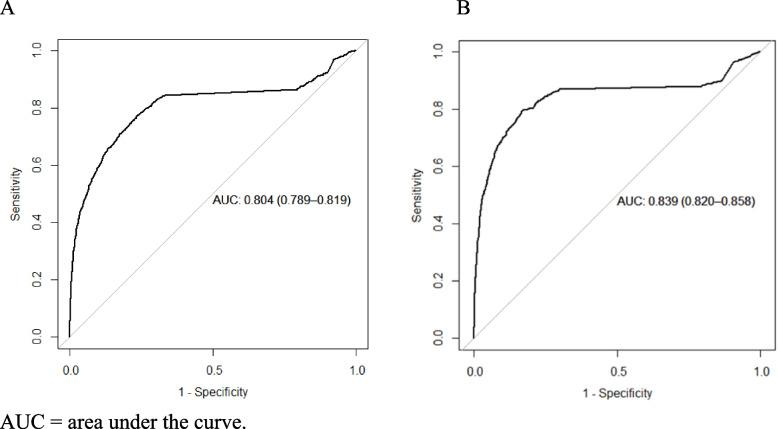
Receiver operator characteristics curve for the optimal stochastic gradient boosting model applied to **A** females and **B** males in the test dataset. AUC, area under the curve

The SGB model showed that PASC was predicted with relatively good accuracy, with an AUC of 0.804 (95% CI: 0.789–0.819) for females and 0.839 (0.820–0.858) for males; see Fig. 1A and B, respectively. Thus, there were 80% correct predictions in females and 84% in males.

The SGB model showed good predictive ability. As seen in Table 2, for female patients, the model identified 5863 out of 6210 females not to have PASC. It also correctly identified 1187 out of 2993 females with PASC. This means that 95% of the females with no PASC diagnoses were correctly identified, and 40% of those diagnosed with PASC. For males, the model identified 3505 out of 3678 males not to have PASC, correctly classifying 95%. Furthermore, it identified 678 out of 1398 males with PASC, in total 48% of those who were diagnosed.

### Variable importance

The diagnoses and prescribed medications that were significantly predictive in PASC had an NRI ≥ 1% and are presented in Table 3 for females and Table 4 for males. All variables included in the machine learning model are presented in Additional file: Tables 1 and 2. The number of variables surpassing the 1% NRI threshold differed slightly between sexes, with 17 variables for females and 15 for males. This reflects differences in the predictive relevance of certain diagnoses and prescribed medications, emphasizing the importance of sex-stratified analyses in understanding PASC,

**Table 3:** The 17 variables with highest NRIs for predicting presence of PASC among females, based on the optimal stochastic gradient boosting model with 15,377 trees, together with OR_ME_ during the year before PASC diagnosis

**Females**			
**ICD- 10 or ATC code**	**Description**	**NRI (%)**	**OR**_**ME**_
U071 and U072	COVID- 19—in hospitalized patients	16.1	18.8
R53	Malaise and fatigue	14.5	4.6
G933	Post-viral and related fatigue syndromes	10.1	21.1
R06	Dyspnea	8.4	6.2
J01, J02, J03, J06 and J20	Upper respiratory tract infections	5.9	3.2
R43	Disturbances of smell and taste	4.4	28.9
B34	Viral infection of unspecified site	3.1	3.5
R03 A and R03B (ATC)	Adrenergics, inhalants and other drugs for obstructive airway diseases, inhalants	2.8	1.8
R00	Tachycardia	2.6	3.0
F43	Reaction to severe stress, and adjustment orders	2.3	2.1
R05	Cough	2.1	2.3
R51	Headache	2.0	2.6
Z86	Personal history of certain other diseases	1.7	5.6
R50	Fever	1.6	4.4
F41	Anxiety disorders	1.6	1.6
G03 A (ATC)	Hormonal contraceptives for systemic use	1.3	2.3
Z02	Encounter for administrative examination	1.0	2.2

*NRI* normalized relative influence, *PASC* post-acute sequelae of COVID- 19, *OR*_*ME*_ odds ratio for marginal effects, *ICD- 10* International Classification of Diseases Tenth Revision, *ATC* Anatomical Therapeutic Chemical

**Table 4:** The 15 variables with highest NRIs for predicting presence of PASC among males, based on the optimal stochastic gradient boosting model with 11,221 trees, together with OR_ME_ during the year before PASC diagnosis

**Males**			
ICD- 10 or ATC code	Description	NRI (%)	OR_ME_
U07, U08, U09, U11 and U12	COVID- 19—in hospitalized patients	41.7	31.6
R53	Malaise and fatigue	11.5	7.9
G933	Post-viral and related fatigue syndromes	6.4	28.4
R06	Dyspnea	4.9	6.5
R05	Cough	4.1	4.4
J01, J02, J03, J06 and J20	Upper respiratory tract infections	3.5	3.4
R00	Tachycardia	2.5	5.2
B34	Viral infection of unspecified site	2.1	3.3
R03 A and R03B (ATC)	Adrenergics, inhalants and other drugs for obstructive airway diseases, inhalants	1.5	1.8
R51	Headache	1.4	3.1
F43	Reaction to severe stress and adjustment orders	1.2	2.6
R05 (ATC)	Cough and cold preparations	1.2	2.2
J45 and J46	Asthma	1.1	2.0

*NRI* normalized relative influence, *PASC* post-acute sequelae of COVID- 19, *OR*_*ME*_ odds ratio for marginal effects, *ICD- 10* International Classification of Diseases Tenth Revision, *ATC* Anatomical Therapeutic Chemical

Among females, 125 diagnoses and 69 medications showed an NRI of more than 0%, with 17 of these diagnoses and drugs combined having a relative influence of over 1%. The five diagnoses with the highest NRIs were COVID- 19 with inpatient care (hospitalization) at 41.7%, malaise and fatigue at 14.5%, post-viral and related fatigue syndromes at 10.1%, dyspnea at 8.4%, and upper respiratory tract infections at 5.9%. top prescribed medications with the highest NRIs were adrenergics, inhalants and other drugs for obstructive airway diseases, and inhalant medicines at 2.8%, and hormonal contraceptives for systemic use at 1.3%.

Among males, 78 diagnoses and 52 medications showed an NRI above 0%, and 15 of these had a relative important influence of over 1%. The five diagnoses with the highest NRIs were COVID- 19 with inpatient care at 41.7%, malaise and fatigue at 11.5%, post-viral and related fatigue syndromes at 6.4%, dyspnea at 8.4%, and cough at 4.1%. The prescribed medications with the highest NRIs were adrenergics, inhalants and other drugs for obstructive airway diseases, and inhalant medicines at 1.5%.

### Marginal effects

The results for the sex-stratified statistical models showed that the top five diagnoses with the highest NRIs had an OR_ME_ above 1. For females, these diagnoses were disturbances of smell and taste (OR_ME_ 28.9), post-viral and related fatigue syndromes (OR_ME_ 21.1), COVID- 19 in inpatient care (OR_ME_ 18.8), dyspnea (OR_ME_ 6.2), and personal history of other diseases (OR_ME_ 5.6) (Table 3). For males, the top five diagnoses were COVID- 19 with inpatient care (OR_ME_ 31.6), post-viral and related fatigue syndromes (OR_ME_ 28,4), malaise and fatigue (OR_ME_ 7.9), dyspnea (OR_ME_ 6.5), and tachycardia (OR_ME_ 5.2) (Table 4).

## Discussion

In the present study, with a large study population of subjects registered at primary health care centers (PHCCs)s in Region Stockholm, data was analyzed included diagnoses recorded at PHC consultation and prescribed medication from the year before PASC diagnosis. Our findings suggest that the machine learning SGB models have promising potential for identifying subjects at risk of PASC and uncovering associations between various factors and PASC. As this is an observational study, these results should be interpreted as identifying patterns and correlations rather than establishing causal relationships. We found that females were at higher risk of being diagnosed with PASC than males. Previous hospitalization due to acute COVID- 19 was strongly associated with an increased risk of PASC in both sexes. Several diagnoses from primary care physicians were significantly linked to higher risk of PASC. These included post-viral and related fatigue syndrome, symptom diagnoses, such as malaise, fatigue, dyspnea, impaired smell and taste, tachycardia, cough and headache, reactions to acute and severe stress in both sexes, as well as anxiety in females and asthma in males. Among the prescribed medications, adrenergic inhalants and other inhalants for obstructive airway diseases in both sexes, and hormonal medication in females, were also linked to higher risk of PASC.

As we hypothesized, the presented method could be applied as a prediction tool for PASC. Our presented machine learning model could be clinically relevant, as it can support diagnostic of PASC in primary care, as long as there are no biomarkers or objective diagnostic tests for PASC. Our findings of several PASC predictors may provide some insight into PASC etiology, which is currently unknown. The model is robust and reproducible, and when more data sources are identified, it can be retrained in further studies.

We found that prior hospitalization due to COVID-19 was the strongest predictor associated with PASC in both sexes. However, a recent meta-analysis suggests that association between hospitalization and PASC is still inconclusive due to studies including mixed cohorts, with both hospitalized and non-hospitalized patients, and some not having data on whether individuals were treated at intensive care [37]. This discrepancy might be attributed to that the meta-analysis using only statistical linear models, while our analysis allowed us to capture linear and nonlinear relationships and interactions among predictors that are typical for a multifactorial condition like PASC. In fact, follow-up studies of PASC have primarily been initiated with hospitalized patients, stating the risk of PASC increased with the severity of initial COVID-19 infection [28, 38, 39]. A meta-analysis showed that long hospitalization and having received intensive care more than doubled the risk of PASC [40]. Even if the association among hospitalized patients is complicated, as the studies usually have not considered post-intensive care syndrome as a differential diagnosis to PASC [41].

We demonstrated that a symptom diagnosis of malaise and fatigue or post-viral fatigue syndrome (ICD-10 G933), which encompasses myalgic encephalomyelitis/chronic fatigue syndrome (ME/CFS), were strongly associated with the increased the risk of PASC in both sexes. PASC and ME/CFS share major symptoms, including chronic fatigue. Diagnostics are based on the presence and duration of symptoms and exclusion of other causes [42]. Previous cross-sectional studies have suggested that 43–58% of PASC patients meet the ME/CFS diagnostic criteria [43–45]. In those studies, ME/CFS and PASC were more prevalent in the non-hospitalized female population. The underlying mechanism for this observation is not fully understood but are thought to involve a combination of sex-specific factors including stronger immune response in females after infection and hormonal influences [45]. The clinical similarities between ME/CFS and PASC allow us to suggest a multifactorial etiology and pathobiology, including a preceding viral illness, increase in inflammation cytokines, neuroinflammation, mitochondrial dysfunction, and alteration in natural killer cell function [42].

We also found that symptom diagnoses, such as dyspnea, disturbances in smell and taste, tachycardia, cough, and headache, as well as upper respiratory tract infections, were predictors of PASC in both sexes. Similar respiratory symptomatology is well-described in other respiratory viral syndromes, including those from severe acute respiratory syndrome, respiratory syncytial virus, and influenza [46, 47]. Further, SARS-CoV-2 is primarily a respiratory virus, meaning that long-term respiratory symptoms are not surprising, and associations have been shown in several previous studies [4, 13–15, 48, 49]. Other symptoms, including tachycardia and headache, have been reported as symptoms of PASC and are included in WHO’s case definition [2].

Furthermore, our results showed an association between PASC and reactions to acute and severe stress in both sexes, and also anxiety in females. In line with this, a large epidemiological study showed that pre-infection psychological distress, including depression, anxiety, and loneliness, was a risk factor for PASC [50]. Mounting evidence supports the link between psychiatric disorders and immune system dysregulation, suggesting that they are closely intertwined and possibly power each other in a bidirectional loop [51, 52].

Our results showing that females appeared to be at greater risk than males of developing PASC, with a ratio 1.8:1 being consistent with prior research, including meta-analyses [38, 53]. Those studies showed that the mortality and hospitalization rates due to COVID- 19 were lower in females than males. It was suggested that sociodemographic aspects played a role in sex differences of persistent symptoms following COVID-19 [47]. Female patients are more likely than male patients to seek healthcare for both physical and mental symptoms [54]. Beyond sociodemographic aspects, biological factors, including hormones and immune responses, may also influence the higher reported prevalence of PASC among females. Female immune systems exhibit stronger innate and adaptive responses compared to males, which could lead to greater immune activation and a higher likelihood of prolonged post-viral symptoms. Sex hormones have been shown to modulate immune responses, potentially leading to differences in inflammation, autoimmunity, and tissue repair following infection [55]. In this study, we also demonstrated that prescribed female sex hormones (ATC code G03), including contraceptives and hormonal replacement therapy, were associated with an increased risk of PASC. Studies on associations between hormonal drugs and PASC are scarce. A previous study based on a nationwide internet-based survey among Swedish women showed that they self-reported feeling that access to contraceptives decreased during the pandemic and that there was an overall decrease in current use of contraceptives compared with pre-pandemic levels [56]. Therefore, in light of other results, our findings suggest that receiving female sex hormones might play a role in PASC etiology.

Another finding was that adrenergic inhalants and other inhalants for obstructive airway diseases were associated with PASC. Furthermore, we showed that asthma contributed to the prediction of PASC in males, but not in females, in concordance with another study [57]. In addition, meta-analyses have reported contradictory results regarding susceptibility to COVID-19 in patients with asthma [58–60]. There are indications that age and severity of disease in asthma affects the outcome of COVID-19 [60, 61]. There is a lack of studies on post-COVID- 19 status in relation to asthma. In a UK-based survey in patients with asthma, 10.5% reported COVID- 19, of these, 56% reported having PASC [62].

In contrast to other researchers, we did not find that other chronic diseases—such as obesity, type 2 diabetes, chronic obstructive pulmonary disease, or ischemic heart diseases—were risk factors for PASC [27, 28, 40, 58]. This might be because we based our analysis only on diagnostic codes registered during the year before the index date, and this was during the pandemic, when many non-urgent healthcare and follow-up visits were postponed. Thus, diagnosing of chronic conditions might have been limited. Nor did we see that dispensing of medications for these conditions, which we in this study saw as a proxy for chronic conditions, was associated with PASC. This was observed despite the fact that a Swedish study showed that there was an increase in the volume of dispensed medication early in the pandemic, possibly due to individuals with chronic disease having decided to dispense extra supplies of medication in case of lockdown [63]. Furthermore, in Sweden, a large proportion (70%) of the population with chronic disease is diagnosed and followed up annually in primary care [64].

The clinical relevance of using SGB, as we did, is that it enables analyses of large amounts of complex data and therefore identify previously unknown relationships in females and males separately. The rationale for dividing the population into female and male patients was based on known sex differences in PASC presentation and symptoms [38, 53]. The model demonstrated strong predictive performance, accurately identifying the majority of patients without PASC.

Although the pandemic has ended, COVID-19 is still circulating and the cumulative incidence of PACS is still substantial [65]. It is therefore important to continue studies on the consequences of COVID-19, in order to learn for future outbreaks. This knowledge could potentially assist health care personnel in prioritizing patients when allocating limited healthcare resources.

For future directions, integrating this method into clinical workflows will require external validation in other Swedish and international populations. Additionally, in future studies, incorporating data beyond February 2022 will help assess the model’s robustness across different pandemic phases. Future studies should also explore potential biomarkers, healthcare utilization, cost-effectiveness, resource requirements, and practical strategies for integrating such tools into existing healthcare systems. Furthermore, from a clinical perspective incorporating detailed hospitalization data, such as length of stay, use of intensive care, or specific treatments received, would help clarify the relationship between acute illness severity and PASC risk.

### Strengths and limitations

The main strength of this study is the large size of population-based data from primary care settings, where studies are scarce. The study included individuals of different ages and both sexes from both urban and rural areas within Region Stockholm, Sweden. The region represents a fast growing population of 2.5 million [32], amounting to approximately 25% of entire population in Sweden [66]. Sweden has a unique infrastructure with register databases. However, primary care data are only registered locally. Our dataset is unique and included all ICD-10 diagnoses recorded by primary care physicians from the VAL databases. Unlike previous studies, which primarily focused on hospitalized cohorts or secondary care data, our work incorporates primary care data and prescribed medications, offering a broader perspective on PASC predictors. Additionally, we used stochastic gradient boosting (SGB), which allows for the inclusion of numerous variables and the modeling of complex interactions between them. This approach enables the identification of both known and novel predictors of PASC, such as hormonal medication in female patients, which were not highlighted in earlier studies. By focusing on primary care data, our methodology provides a more comprehensive and nuanced understanding of PASC predictors, advancing beyond existing approaches that are limited to secondary care settings.

Another strength of our study is the use of the SGB method, where all included variables, such as diagnoses and medications, were incorporated into the model along with their interactions with each other. The SGB model can be considered as adjusted for hundreds of variables, enabling it to account for complex, non-linear relationships and interactions that might act as confounders. While we stratified by age and sex, which are known key factors in PASC, we recognize that other potential confounders—such as socioeconomic status, vaccination status, and pre-existing comorbidities—were not explicitly adjusted for in this study.

The use of SGB allows the inclusion of individuals with missing data, mitigating the impact of exclusion of patients with incomplete records. However, as with any registry-based study, underreporting of diagnoses or healthcare contacts is a potential limitation. For example, individuals who only occasionally seek healthcare may have fewer registered data points, which could impact predictor completeness. Despite this, the robustness of the SGB model ensures that the analysis can incorporate such cases. Our model demonstrated strong predictive performance, in identifying over 90% of patients without PASC. Its accuracy in identifying patients with PASC was 40% of the female and 48% male patients classified at risk of PASC actually had PASC, indicating that those identified by the model are at high risk of PASC. These findings highlight the model’s potential for clinical use, while also underscoring the need for further refinement.

There are several limitations in this study. One is that general practitioners may not document all diagnoses, particularly symptom diagnoses, presented during healthcare visit [67]. Instead, they may focus on the specific reasons for each particular visit, which should be taken into consideration when interpreting our results. Furthermore, conditions such as chronic obstructive pulmonary disease and obesity are known to be underdiagnosed. Further, some of the diagnoses associated with PASC in our study can be difficult to interpret, such as ICD code Z86.1: Personal history of COVID-19, which was used in Sweden only during the period June 1, 2020 to December 31, 2021 [68].

Another limitation of our study is that we do not differentiate between hospitalized patients and patients treated in intensive care. The strong association between prior hospitalization due to COVID- 19 and PASC likely reflects the heightened risk associated with more severe acute illness. However, our inability to differentiate between levels of care intensity during hospitalization limits our ability to fully interpret this finding.

Furthermore, the data were based on diagnostic codes (ICD-10) and prescription drug codes (ATC). We were not able to assess some potential confounders that could influence PASC predictors including other sociodemographic factors except for age and sex, self-reported lifestyle factors such as smoking habits, symptom severity, or the potential impact on quality of life. We did not evaluate the impact of vaccines, which are known to play a protective role against PASC [40]. Furthermore, the study did not assess the effect of therapeutics, such as Paxlovid, on the likelihood of PASC diagnosis, nor did we evaluate the association with COVID- 19 reinfection or consider that the controls could be diagnosed with PASC outside the timeframe of this study. This residual confounding was mitigated in the study by using SGB modeling, which allows for the inclusion of multiple variables and complex interactions, improving the model’s ability to account for unmeasured confounders.

Another weakness is that no specific ICD- 10 code for PASC is available, possibly resulting in variations of usage of the diagnostic code U09.9 between physicians and healthcare facilities. For example, the highest usage of the diagnosis code U09.9 among Swedish metropolitan areas was reported in the Region Stockholm, where this study was conducted [17]. Thus, there may have been misclassification and underreporting of PASC in other regions of Sweden. As both the diagnosis code and the syndrome PASC are novel, the use of the diagnosis will likely have evolved during the study period. Clinicians may be more likely to report this diagnosis now than at the beginning of the study period. Future research should explore these acknowledge biases to validate findings and further refine predictive models in later cohorts.

## Conclusions

This study demonstrated that the SGB model can identify associations between PASC and registered diagnoses, as well as prescribed medications, during the year before a PASC diagnosis. Known risk factors, such as previous hospitalization due to COVID-19, respiratory, neurological, and cognitive symptoms, and the use of inhalation medicines in both sexes, as well as asthma in male patients, were verified. Additionally, novel predictors, such as hormonal medication in female patients, were identified and warrant further investigation. While these findings highlight the potential of machine learning for exploring PASC predictors, the model requires external validation and further refinement before it can be considered for implementation in clinical settings.

## Supplementary Information

Additional file 1: Table S1. [Merged ICD- 10 codes]. Table SS. [Medication of interest as ATC-codes, in high resolution or merged].

## Data Availability

No datasets were generated or analysed during the current study.

## Abbreviations

PASC: Post-acute sequelae of COVID- 19
NRI: Normalized relative influence
OR_ME_: Odds ratios of marginal effects
COVID- 19: Coronavirus disease 2019
SARS-CoV- 2: Severe acute respiratory syndrome coronavirus 2
SGB: Stochastic gradient boosting
PHC: Primary healthcare
PHCC: PHC centers
ICD- 10: International Classification of Diseases, Tenth Revision
ATC code: Anatomical Therapeutic Chemical code
ROC curve: Receiver operator characteristics curve
AUC: Area under the curve
ME/CFS: Myalgic encephalomyelitis/chronic fatigue syndrome

## References

[1] Rajan S, Khunti K, Alwan N, Steves C, MacDermott N, Morsella A, et al. In the wake of the pandemic: preparing for long COVID. Copenhagen (Denmark): European Observatory Policy Briefs; 2021. 33877759

[2] WHO. A clinical case definition of post COVID-19 condition by a Delphi consensus, https://www.who.int/publications/i/item/WHO-2019-nCoV-Post_COVID-19_condition-Clinical_case_definition-2021.1 Last seen 20231013. 2021. 10.1016/S1473-3099(21)00703-9 PMC8691845 34951953

[3] O’Mahoney LL, Routen A, Gillies C, Ekezie W, Welford A, Zhang A, et al. The prevalence and long-term health effects of Long Covid among hospitalised and non-hospitalised populations: a systematic review and meta-analysis. EClinicalMedicine. 2023;55:101762. 36474804 10.1016/j.eclinm.2022.101762 PMC9714474

[4] Kisiel MA, Lee S, Malmquist S, Rykatkin O, Holgert S, Janols H, et al. Clustering analysis identified three long COVID phenotypes and their association with general health status and working ability. J Clin Med. 2023;12(11):3617. 37297812 10.3390/jcm12113617 PMC10253616

[5] Kisiel MA, Lee S, Janols H, Faramarzi A. Absenteeism Costs Due to COVID-19 and their predictors in non-hospitalized patients in sweden: a poisson regression analysis. Int J Environ Res Public Health. 2023;20(22):7052. 37998283 10.3390/ijerph20227052 PMC10671172

[6] Kisiel MA, Nordqvist T, Westman G, Svartengren M, Malinovschi A, Janols H. Patterns and predictors of sick leave among Swedish non-hospitalized healthcare and residential care workers with Covid-19 during the early phase of the pandemic. PLoS ONE. 2021;16(12):e0260652. 34882720 10.1371/journal.pone.0260652 PMC8659339

[7] Katsoularis I, Fonseca-Rodriguez O, Farrington P, Lindmark K, Connolly AF. COVID-19 and myocardial infarction - Authors’ reply. Lancet. 2021;398(10315):1964. 34838176 10.1016/S0140-6736(21)02320-5 PMC8616566

[8] Katsoularis I, Fonseca-Rodriguez O, Farrington P, Jerndal H, Lundevaller EH, Sund M, et al. Risks of deep vein thrombosis, pulmonary embolism, and bleeding after covid-19: nationwide self-controlled cases series and matched cohort study. BMJ. 2022;377: e069590. 35387772 10.1136/bmj-2021-069590 PMC8984137

[9] Crook H, Raza S, Nowell J, Young M, Edison P. Long covid-mechanisms, risk factors, and management. BMJ. 2021;374:n1648. 34312178 10.1136/bmj.n1648

[10] Greenhalgh T, Sivan M, Perlowski A, Nikolich JZ. Long COVID: a clinical update. Lancet. 2024;404(10453):707–24. 39096925 10.1016/S0140-6736(24)01136-X

[11] Sigfrid L, Drake TM, Pauley E, Jesudason EC, Olliaro P, Lim WS, et al. Long covid in adults discharged from UK hospitals after Covid-19: a prospective, multicentre cohort study using the ISARIC WHO Clinical Characterisation Protocol. Lancet Reg Health Eur. 2021;8:100186. 34386785 10.1016/j.lanepe.2021.100186 PMC8343377

[12] Fernandez-de-Las-Penas C, Raveendran AV, Giordano R, Arendt-Nielsen L. Long COVID or post-COVID-19 condition: past, present and future research directions. Microorganisms. 2023;11(12):2959. 38138102 10.3390/microorganisms11122959 PMC10745830

[13] Sudre CH, Murray B, Varsavsky T, Graham MS, Penfold RS, Bowyer RC, et al. Attributes and predictors of long COVID. Nat Med. 2021;27(4):626–31. 33692530 10.1038/s41591-021-01292-y PMC7611399

[14] Al-Aly Z, Bowe B, Xie Y. Long COVID after breakthrough SARS-CoV-2 infection. Nat Med. 2022;28(7):1461–7. 35614233 10.1038/s41591-022-01840-0 PMC9307472

[15] Lopez-Leon S, Wegman-Ostrosky T, Perelman C, Sepulveda R, Rebolledo PA, Cuapio A, Villapol S. More than 50 long-term effects of COVID-19: a systematic review and meta-analysis. Sci Rep. 2021;11(1):16144. 10.1038/s41598-021-95565-8 PMC8352980 34373540

[16] Kisiel MA, Janols H, Nordqvist T, Bergquist J, Hagfeldt S, Malinovschi A, et al. Predictors of post-COVID-19 and the impact of persistent symptoms in non-hospitalized patients 12 months after COVID-19, with a focus on work ability. Ups J Med Sci. 2022;127:10–48101. 10.48101/ujms.v127.8794 PMC9383047 35991464

[17] Ollila HM F-Rodríguez O, Caspersen IH, et al. How do clinicians use post-COVID syndrome diagnosis? Analysis of clinical features in a Swedish COVID-19 cohort with 18 months’ follow-up: a national observational cohort and matched cohort study. BMJ Public Health. 2024;2:e000336. 10.1136/bmjph-2023-000336. 10.1136/bmjph-2023-000336 PMC11816610 40018228

[18] Al-Aly Z, Davis H, McCorkell L, Soares L, Wulf-Hanson S, Iwasaki A, et al. Long COVID science, research and policy. Nat Med. 2024;30(8):2148–64. 39122965 10.1038/s41591-024-03173-6

[19] Wandell P, Carlsson AC, Wierzbicka M, Sigurdsson K, Arnlov J, Eriksson J, et al. A machine learning tool for identifying patients with newly diagnosed diabetes in primary care. Prim Care Diabetes. 2024;18(5):501–5. 38944562 10.1016/j.pcd.2024.06.010

[20] Ainiwaer A, Hou WQ, Qi Q, Kadier K, Qin L, Rehemuding R, et al. Deep learning of heart-sound signals for efficient prediction of obstructive coronary artery disease. Heliyon. 2024;10(1):e23354. 38169906 10.1016/j.heliyon.2023.e23354 PMC10758826

[21] Abdar M, Ksiazek W, Acharya UR, Tan RS, Makarenkov V, Plawiak P. A new machine learning technique for an accurate diagnosis of coronary artery disease. Comput Methods Programs Biomed. 2019;179: 104992. 31443858 10.1016/j.cmpb.2019.104992

[22] Ainiwaer A, Hou WQ, Kadier K, Rehemuding R, Liu PF, Maimaiti H, et al. A Machine Learning Framework for Diagnosing and Predicting the Severity of Coronary Artery Disease. Rev Cardiovasc Med. 2023;24(6): 168. 39077543 10.31083/j.rcm2406168 PMC11264126

[23] Nemlander E, Ewing M, Abedi E, Hasselstrom J, Sjovall A, Carlsson AC, et al. A machine learning tool for identifying non-metastatic colorectal cancer in primary care. Eur J Cancer. 2023;182:100–6. 36758474 10.1016/j.ejca.2023.01.011

[24] Nemlander E, Ewing M, Carlsson AC, Rosenblad A. Transforming early cancer detection in primary care: harnessing the power of machine learning. Oncoscience. 2023;10:20–1. 37303966 10.18632/oncoscience.578 PMC10254750

[25] Wynants L, Van Calster B, Collins GS, Riley RD, Heinze G, Schuit E, et al. Prediction models for diagnosis and prognosis of covid-19: systematic review and critical appraisal. BMJ. 2020;369:m1328. 32265220 10.1136/bmj.m1328 PMC7222643

[26] Ahmad I, Amelio A, Merla A, Scozzari F. A survey on the role of artificial intelligence in managing Long COVID. Front Artif Intell. 2023;6:1292466. 38274052 10.3389/frai.2023.1292466 PMC10808521

[27] Pfaff ER, Girvin AT, Bennett TD, Bhatia A, Brooks IM, Deer RR. Identifying who has long COVID in the USA: a machine learning approach using N3C data. The Lancet Digital Health. 2022;4(7):E532–41. 35589549 10.1016/S2589-7500(22)00048-6 PMC9110014

[28] Hill EL, Mehta HB, Sharma S, Mane K, Singh SK, Xie C, et al. Risk factors associated with post-acute sequelae of SARS-CoV-2: an N3C and NIH RECOVER study. BMC Public Health. 2023;23(1):2103. 37880596 10.1186/s12889-023-16916-w PMC10601201

[29] Stasenko SV, Kovalchuk AV, Eremin EV, Drugova OV, Zarechnova NV, Tsirkova MM, et al. Using machine learning algorithms to determine the post-COVID state of a person by their rhythmogram. Sensors (Basel). 2023;23(11):5272. 37299999 10.3390/s23115272 PMC10256087

[30] Frondelius T, Atkova I, Miettunen J, Rello J, Vesty G, Chew HSJ, et al. Early prediction of ventilator-associated pneumonia with machine learning models: a systematic review and meta-analysis of prediction model performance(✰). Eur J Intern Med. 2024;121:76–87. 37981529 10.1016/j.ejim.2023.11.009

[31] Nemlander E, Rosenblad A, Abedi E, Ekman S, Hasselstrom J, Eriksson LE, et al. Lung cancer prediction using machine learning on data from a symptom e-questionnaire for never smokers, formers smokers and current smokers. PLoS ONE. 2022;17(10): e0276703. 36269746 10.1371/journal.pone.0276703 PMC9586380

[32] https://www.regionstockholm.se/nyheter/2024/09/befolkningsokningen-bedoms-bli-mindre-visar-arets-prognos/. 2024. February 24, 2025.

[33] VAL-databaserna – Region Stockholm Stockholm: Centrum för epidemiologi och samhällsmedicin, Region Stockholm; 2024. https://www.folkhalsokollen.se/datakallor/val-databaserna/. Accessed 3 May 2024.

[34] Friedman JH. Greedy Function Approximation: A Gradient Boosting Machine. The Annals of Statistics. 2001;29(No. 5 (Oct., 2001)):1189-232 (44 pages).

[35] Norrman A, Hasselstrom J, Ljunggren G, Wachtler C, Eriksson J, Kahan T, et al. Predicting new cases of hypertension in Swedish primary care with a machine learning tool. Prev Med Rep. 2024;44:102806. 39091569 10.1016/j.pmedr.2024.102806 PMC11292513

[36] Team RC. R: A language and environment for statistical computing. R Foundation for Statistical Computing, Vienna, Austria. 2016. http://www.R-project.org/.

[37] Fernandez-de-Las-Penas C, Notarte KI, Macasaet R, Velasco JV, Catahay JA, Ver AT, et al. Persistence of post-COVID symptoms in the general population two years after SARS-CoV-2 infection: A systematic review and meta-analysis. J Infect. 2024;88(2):77–88. 38101521 10.1016/j.jinf.2023.12.004

[38] Notarte KI, de Oliveira MHS, Peligro PJ, Velasco JV, Macaranas I, Ver AT, et al. Age, sex and previous comorbidities as risk factors not associated with SARS-CoV-2 infection for long COVID-19: a systematic review and meta-analysis. J Clin Med. 2022;11(24):7314. 36555931 10.3390/jcm11247314 PMC9787827

[39] Sahu AK, Mathew R, Aggarwal P, Nayer J, Bhoi S, Satapathy S, et al. Clinical determinants of severe COVID-19 disease - a systematic review and meta-analysis. J Glob Infect Dis. 2021;13(1):13–9. 33911447 10.4103/jgid.jgid_136_20 PMC8054797

[40] Tsampasian V, Elghazaly H, Chattopadhyay R, Debski M, Naing TKP, Garg P, et al. Risk Factors Associated With Post-COVID-19 condition: a systematic review and meta-analysis. JAMA Intern Med. 2023;183(6):566–80. 36951832 10.1001/jamainternmed.2023.0750 PMC10037203

[41] Bangash MN, Owen A, Alderman JE, Chotalia M, Patel JM, Parekh D. COVID-19 recovery: potential treatments for post-intensive care syndrome. Lancet Respir Med. 2020;8(11):1071–3. 33058770 10.1016/S2213-2600(20)30457-4 PMC7550044

[42] Komaroff AL, Lipkin WI. ME/CFS and Long COVID share similar symptoms and biological abnormalities: road map to the literature. Front Med (Lausanne). 2023;10:1187163. 37342500 10.3389/fmed.2023.1187163 PMC10278546

[43] Jason LA, Dorri JA. ME/CFS and Post-Exertional Malaise among Patients with Long COVID. Neurol Int. 2022;15(1):1–11. 36648965 10.3390/neurolint15010001 PMC9844405

[44] Mancini DM, Brunjes DL, Lala A, Trivieri MG, Contreras JP, Natelson BH. Use of Cardiopulmonary Stress Testing for Patients With Unexplained Dyspnea Post-Coronavirus Disease. JACC Heart Fail. 2021;9(12):927–37. 34857177 10.1016/j.jchf.2021.10.002 PMC8629098

[45] Bonilla H, Quach TC, Tiwari A, Bonilla AE, Miglis M, Yang PC, et al. Myalgic Encephalomyelitis/Chronic Fatigue Syndrome is common in post-acute sequelae of SARS-CoV-2 infection (PASC): Results from a post-COVID-19 multidisciplinary clinic. Front Neurol. 2023;14: 1090747. 36908615 10.3389/fneur.2023.1090747 PMC9998690

[46] Ngai JC, Ko FW, Ng SS, To KW, Tong M, Hui DS. The long-term impact of severe acute respiratory syndrome on pulmonary function, exercise capacity and health status. Respirology. 2010;15(3):543–50. 20337995 10.1111/j.1440-1843.2010.01720.x PMC7192220

[47] Fauroux B, Simoes EAF, Checchia PA, Paes B, Figueras-Aloy J, Manzoni P, et al. The Burden and Long-term Respiratory Morbidity Associated with Respiratory Syncytial Virus Infection in Early Childhood. Infect Dis Ther. 2017;6(2):173–97. 28357706 10.1007/s40121-017-0151-4 PMC5446364

[48] Brunvoll SH, Nygaard AB, Fagerland MW, Holland P, Ellingjord-Dale M, Dahl JA, et al. Post-acute symptoms 3–15 months after COVID-19 among unvaccinated and vaccinated individuals with a breakthrough infection. Int J Infect Dis. 2023;126:10–3. 36375693 10.1016/j.ijid.2022.11.009 PMC9651990

[49] Ballering AV, van Zon SKR, Olde Hartman TC, Rosmalen JGM, Lifelines Corona Research I. Persistence of somatic symptoms after COVID-19 in the Netherlands: an observational cohort study. Lancet. 2022;400(10350):452–61. 35934007 10.1016/S0140-6736(22)01214-4 PMC9352274

[50] Wang S, Quan L, Chavarro JE, Slopen N, Kubzansky LD, Koenen KC, et al. Associations of depression, anxiety, worry, perceived stress, and loneliness prior to infection with risk of post-COVID-19 conditions. JAMA Psychiat. 2022;79(11):1081–91. 10.1001/jamapsychiatry.2022.2640 PMC9453634 36069885

[51] Menard C, Pfau ML, Hodes GE, Russo SJ. Immune and Neuroendocrine Mechanisms of Stress Vulnerability and Resilience. Neuropsychopharmacology. 2017;42(1):62–80. 27291462 10.1038/npp.2016.90 PMC5143517

[52] Bauer ME, Teixeira AL. Inflammation in psychiatric disorders: what comes first? Ann N Y Acad Sci. 2019;1437(1):57–67. 29752710 10.1111/nyas.13712

[53] Mehta HB, Li S, Goodwin JS. Risk factors associated With SARS-CoV-2 infections, hospitalization, and mortality among US nursing home residents. JAMA Netw Open. 2021;4(3):e216315. 33787905 10.1001/jamanetworkopen.2021.6315 PMC8013796

[54] Thompson AE, Anisimowicz Y, Miedema B, Hogg W, Wodchis WP, Aubrey-Bassler K. The influence of gender and other patient characteristics on health care-seeking behaviour: a QUALICOPC study. BMC Fam Pract. 2016;17:38. 27036116 10.1186/s12875-016-0440-0 PMC4815064

[55] Sciarra F, Campolo F, Franceschini E, Carlomagno F, Venneri MA. Gender-specific impact of sex hormones on the immune system. Int J Mol Sci. 2023;24(7):6302. 10.3390/ijms24076302 PMC10094624 37047274

[56] Envall N, Gemzell Danielsson K, Kopp KH. The use and access to contraception in Sweden during the COVID-19 pandemic period. Eur J Contracept Reprod Health Care. 2023;28(5):275–81. 37902288 10.1080/13625187.2023.2260516

[57] Hedberg P, Naucler P. Post-COVID-19 condition after SARS-CoV-2 infections during the omicron surge vs the delta, alpha, and wild type periods in Stockholm, Sweden. J Infect Dis. 2024;229(1):133–6. 37665981 10.1093/infdis/jiad382 PMC10786247

[58] Zhang H, Zang C, Xu Z, et al. Data-driven identification of post-acute SARS-CoV-2 infection subphenotypes. Nat Med. 2023;29:226–35. 10.1038/s41591-022-02116-3 PMC9873564 36456834

[59] Liu S, Cao Y, Du T, Zhi Y. Prevalence of Comorbid Asthma and Related Outcomes in COVID-19: A Systematic Review and Meta-Analysis. J Allergy Clin Immunol Pract. 2021;9(2):693–701. 33309934 10.1016/j.jaip.2020.11.054 PMC7725230

[60] Uruma Y, Manabe T, Fujikura Y, Iikura M, Hojo M, Kudo K. Effect of asthma, COPD, and ACO on COVID-19: a systematic review and meta-analysis. PLoS ONE. 2022;17(11):e0276774. 36318528 10.1371/journal.pone.0276774 PMC9624422

[61] Karlsson Sundbaum J, Konradsen JR, Vanfleteren L, Axelsson Fisk S, Pedroletti C, Sjoo Y, et al. Uncontrolled asthma predicts severe COVID-19: a report from the Swedish National Airway Register. Ther Adv Respir Dis. 2022;16:17534666221091184. 35430944 10.1177/17534666221091183 PMC9019327

[62] Philip KEJ, Buttery S, Williams P, Vijayakumar B, Tonkin J, Cumella A, et al. Impact of COVID-19 on people with asthma: a mixed methods analysis from a UK wide survey. BMJ Open Respir Res. 2022;9(1):e001056. 35027428 10.1136/bmjresp-2021-001056 PMC8762134

[63] Karlsson P, Nakitanda AO, Lofling L, Cesta CE. Patterns of prescription dispensation and over-the-counter medication sales in Sweden during the COVID-19 pandemic. PLoS ONE. 2021;16(8):e0253944. 34388166 10.1371/journal.pone.0253944 PMC8362980

[64] Forslund T WBS. Primärvårdens roll i sjukvårdssystemet. Stockholm: Region Stockholm, Hälso- och sjukvårdsförvaltningen; 2019.

[65] Organization WH. Coronavirus (COVID-19) dashboard. Geneva. 2024. https://covid19.who.int/.

[66] Region S. Europe's most attractive metropolitan region. https://stockholmregion.org/2024.

[67] Ford E, Nicholson A, Koeling R, Tate A, Carroll J, Axelrod L, et al. Optimising the use of electronic health records to estimate the incidence of rheumatoid arthritis in primary care: what information is hidden in free text? BMC Med Res Methodol. 2013;13:105. 23964710 10.1186/1471-2288-13-105 PMC3765394

[68] Statistik om tillstånd efter COVID-19. Primärvård och specialiserad vård. Socialstyrelsen; 2021. Contract No.: Art. No. 2021–6–7495. https://www.socialstyrelsen.se/globalassets/sharepoint-dokument/artikelkatalog/ovrigt/2021-4-7353.pdf.

